# Crystal structure of (*N*,*N*′-ethyl­enebis{3-[2-(3-nitro­phen­yl)hydrazin-1-yl­idene]-4-oxo­pentan-2-iminato})copper(II)–3-[2-(3-nitro­phen­yl)hydrazin-1-yl­idene]pentane-2,4-dione (1/1)

**DOI:** 10.1107/S2056989019005838

**Published:** 2019-05-21

**Authors:** Jan Marten, Wilhelm Seichter, Edwin Weber

**Affiliations:** a TU Bergakademie Freiberg, Leipziger Str. 29, D-09596 Freiberg/Sachsen, Germany

**Keywords:** crystal structure, aryl­hydrazone, Cu^II^ chelate, two-component crystal, mol­ecular layer formation, C—H⋯O hydrogen bonding

## Abstract

In the title 1:1 co-crystal, [Cu(C_24_H_24_N_8_O_6_)]·C_11_H_11_N_3_O_4_, each of the crystal components forms undulating layers which stack alternately along the *b-*axis direction. Mol­ecules of the Cu^II^ complex are connected *via* C—H⋯O hydrogen bonds involving the nitro and keto oxygen atoms, thus forming supra­molecular networks. Mol­ecules of the aryl­hydrazone component are linked by C—H⋯O inter­actions into zigzag strands showing no inter­strand association.

## Abstract 2

The title compound is composed of a bis­(hydrazoniminato)copper(II) complex and the basic hydrazone imine shows a mol­ecular framework with layers each constructed from one of the two components arranged in alternating order. Layer formation is supported by C—H⋯O inter­actions.

## Chemical context   

Hydrazone imines derived from β-diketones and aryl­diazo­nium salts using a Japp–Klingemann route (Phillips, 1959 ▸) have attracted considerable inter­est as precursors of potential anti­diabetic drugs (Garg & Prakash, 1971 ▸; Küçükgüzel *et al.*, 1999 ▸) as well as regarding their particular property of hydrogen bonding (Marten *et al.*, 2007 ▸; Sethukumar & Arul Prakasam, 2010 ▸) and their remarkable behavior in the formation of metal complexes. Transition-metal chelates of the respective hydrazine imines have been described in great numbers (Albert *et al.*, 1997 ▸; Mishra *et al.*, 2000 ▸; Marten *et al.*, 2005 ▸). Preferentially, the chelates with Cu^II^, Co^II^ and Ni^II^ show a tetra­hedrally distorted square N_2_O_2_ coordination environment. In the presence of a di­amine and Ni^II^, related bis(hydrazono­imine) complexes are formed displaying an unusual behavior of the effective magnetic moment at low temperature (Khudina *et al.*, 2007 ▸). A corresponding chelate complex, formed of 3-[2-(3-nitro­phen­yl)hydrazin-1-yl­idene]pen­tane-2,4-dione (Marten *et al.*, 2018 ▸) and bis­(ethyl­enedi­amine)­copper(II) chloride yielded a co-crystal consisting of *N*,*N*′-ethyl­enebis{3-[2-(3-nitro­phen­yl)hydrazine-1-yl­idene]-4-oxo­pentane-2-iminato}copper(II) and 3-[2-(3-nitro­phen­yl)hydrazine-1-yl­idene]pentane-2,4-dione in a 1:1 stoichiometric composition (the title compound), whose crystal structure is reported on herein.
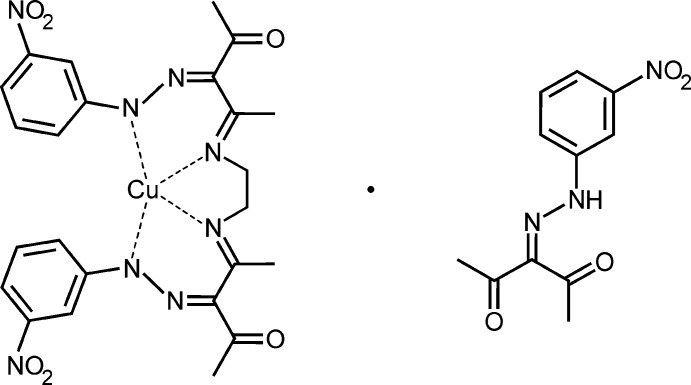


## Structural commentary   

**Figure 1:**
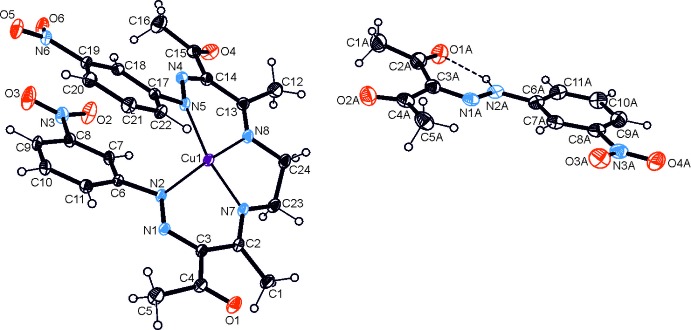
Perspective view of an asymmetric unit of the title co-crystal with atom labeling. Displacement ellipsoids of non-H atoms are shown at the 40% probability level.

The title co-crystal possesses ortho­rhom­bic symmetry (space group *Pbca*) with one mol­ecule of the Cu^II^ complex and one mol­ecule of the aryl­hydrazone in the asymmetric unit. A perspective view is shown in Fig. 1 ▸. The metal center of the complex adopts a tetra­hedrally distorted square coordination environment formed by four nitro­gen atoms (N2, N4, N7, N8) of the ligand. As a result of the tetra­dentate coordination mode and the steric inter­action between the terminal aromatic rings of the ligand, the complex mol­ecule adopts a helical geometry with a distance of 3.384 (4) Å between the benzene ring centroids and a dihedral angle of 10.43 (4)° between the benzene ring planes. The Cu—N distances are 1.940 (2), 1.943 (2), 1.953 (2) and 1.957 (2) Å, the bond angles N7—Cu—N8, N7—Cu—N2, N8—Cu—N5 and N2—Cu—N5 are 86.3 (1), 88.4 (1), 89.1 (1) and 100.4 (1)°, respectively. The nitro groups deviate slightly from the planes of the respective benzene rings, with plane N3/O2/O3 being inclined to benzene ring C6–C11 of 3.8 (1)° and plane N6/O5/O6 being inclined to benzene ring C17–C22 by 5.1 (2)° between the nitro groups and the respective benzene rings. The conformation of the aryl­hydrazone component is nearly identical with that found in the reported structure of this compound (Marten *et al.*, 2018 ▸). The mol­ecule features an intra­molecular N—H⋯O=C inter­action that yields a six-membered hydrogen-bonded ring. The dihedral angle between the mean plane of this ring and the aromatic ring is 7.8 (2)°. The nitro group is tilted at an angle of 5.8 (2)° with respect to the benzene ring.

## Supra­molecular features   

**Figure 2:**
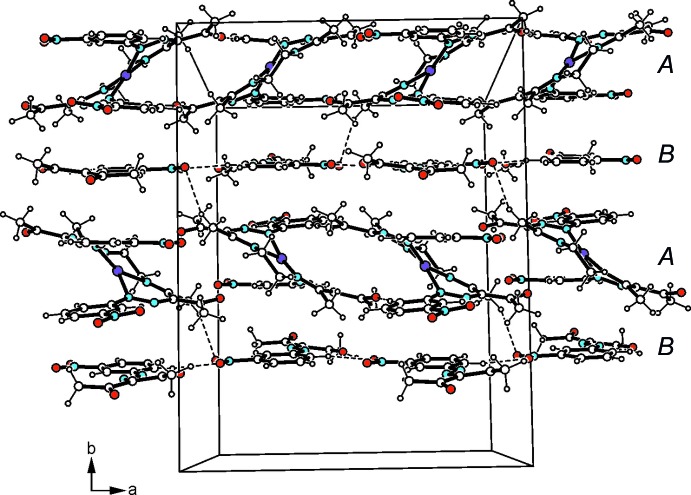
Packing structure of the of the title co-crystal viewed down the crystallographic *c* axis. Dashed lines represent hydrogen bonds.

**Figure 3:**
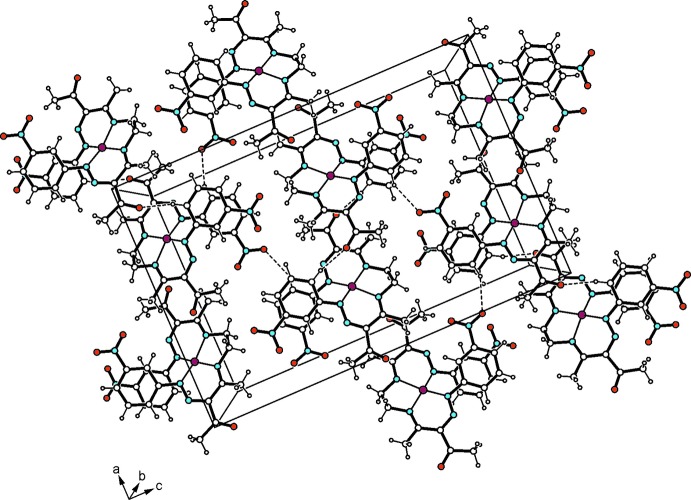
Structure of the Cu^II^ complex layer viewed along the *b* axis. The C—H⋯O inter­actions (Table 1 ▸) are shown as dashed lines.

**Figure 4:**
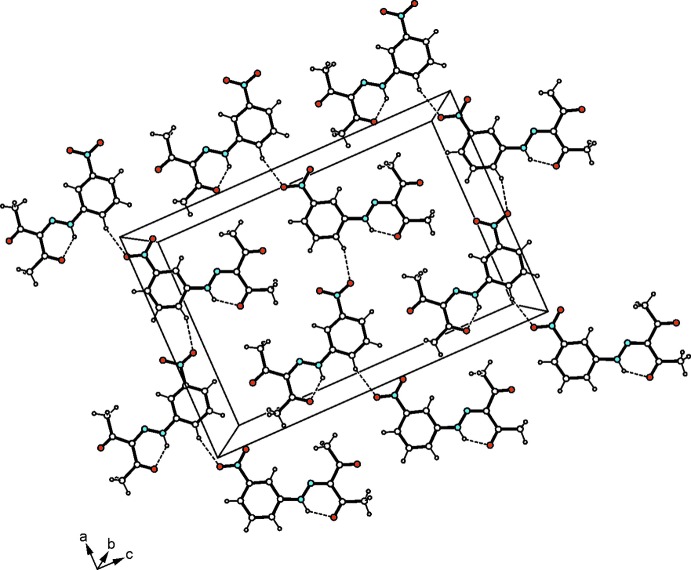
Layer structure of the aryl­hydrazone viewed along the *b* axis. Dashed lines represent the C—H⋯O inter­actions (Table 1 ▸).

In the crystal, the Cu^II^ complexes as well as the aryl­hydrazone mol­ecules form undulating layers extending parallel to the *ac* plane and arranged in an alternating order along the *b*-axis direction (Fig. 2 ▸). Within a layer of complexes, one carbonyl oxygen and one nitro group per mol­ecule inter­act *via* C_arene_—H⋯O hydrogen bonding (Desiraju & Steiner, 1999 ▸), thus generating a supra­molecular network (Table 1 ▸, Fig. 3 ▸). The aryl­hydrazone mol­ecules are connected by means of C_ar­yl_—H⋯O_nitro_ inter­actions to form zigzag-like strands that run along the *a*-axis direction (Table 1 ▸, Fig. 4 ▸). No directed non-covalent bonds are observed between the supra­molecular strands. In the stacking direction, the mol­ecules are linked by C—H⋯O inter­actions involving a nitro oxygen atom of the aryl­hydrazone mol­ecule and a methyl hydrogen of the coordinated ligand.

## Database survey   

A search in the Cambridge Structural Database (CSD, Version 5.38, update February 2017; Groom *et al.*, 2016 ▸) revealed one hit for a crystal structure of a transition-metal complex containing a structurally related ligand species. The complex *N*,*N*′-ethyl­ene-bis­[3-(4-methyl­phen­yl)hydrazono-4-oxo-5,5,6,6,7,7,8,8-octa­fluoro­octane-2-iminato]nickel(II) (JIXQAJ; Khudina *et al.*, 2007 ▸) adopts a helical geometry that resembles that of the title complex. As a result of the presence of two extended fluoro­alkyl moieties, the pattern of inter­molecular non-covalent bonding is dominated by C—H⋯F and F⋯F inter­actions (Reichenbächer *et al.*, 2005 ▸), creating a three-dimensional supra­molecular architecture. Unlike the title structure, in the reported crystal structure of 3-[2-(3-nitro­phen­yl)hydrazine-1-yl­idene]pentane-2,4-dione (Marten *et al.*, 2018 ▸) the mol­ecules are connected *via* C_arene_—H⋯O_nitro_ and C_arene_—H⋯O_keto_ inter­actions giving rise to supra­molecular sheets.

## Synthesis and crystallization   

A solution containing 3-[2-(3-nitro­phen­yl)hydrazine-1-yl­idene]pentane-2,4-dione and bis­(ethyl­enedi­amine)­copper(II) chloride in *n*-butanol was heated for several hours. After cooling and storing the reaction solution, blue-colored crystals could be isolated which turned out to consist of the title compound.

## Refinement   

Crystal data, data collection and structure refinement details are summarized in Table 2 ▸. The NH H atom of 3-[2-(3-nitrophenyl)-hydrazine-1-ylidene]pentane-2,4-dione was located in a difference-Fourier map and freely refined. The C-bound and N-bound H atoms were included in the model in calculated positions and refined as riding atoms: C—H = 0.95–0.99 Å with *U*_iso_(H) = 1.5*U*_eq_(C) for methyl and *U*_iso_(H) = 1.2*U*_eq_(C) for other H atoms.

## Supplementary Material

Crystal structure: contains datablock(s) I. DOI: 10.1107/S2056989019005838/yk2120sup1.cif

Structure factors: contains datablock(s) I. DOI: 10.1107/S2056989019005838/yk2120Isup2.hkl

CCDC reference: 1912679

Additional supporting information:  crystallographic information; 3D view; checkCIF report

## Figures and Tables

**Table 1:** Hydrogen-bond geometry (Å, °)

*D*—H⋯*A*	*D*—H	H⋯*A*	*D*⋯*A*	*D*—H⋯*A*
C11*A*—H11*A*⋯O4*A* ^i^	0.95	2.43	3.320 (3)	156
N2*A*—H2*A*⋯O1*A*	0.90 (1)	1.81 (2)	2.531 (3)	135 (3)
C22—H22⋯O1^ii^	0.95	2.27	3.207 (3)	169
C16—H16*A*⋯O5^iii^	0.98	2.63	3.532 (3)	154
C12—H12*B*⋯O3*A* ^iv^	0.98	2.53	3.413 (3)	151
C11—H11⋯O3^v^	0.95	2.50	3.350 (3)	150
C5—H5*C*⋯O3^v^	0.98	2.65	3.426 (3)	137
C1—H1*B*⋯O3*A* ^vi^	0.98	2.58	3.490 (3)	154
C1—H1*B*⋯O1	0.98	2.34	2.842 (3)	111

Symmetry codes: (i) 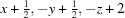; (ii) 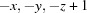; (iii) 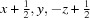; (iv) 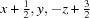; (v) 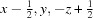; (vi) 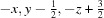.

**Table 2:** Experimental details

Crystal data	
Chemical formula	[Cu(C_24_H_24_N_8_O_6_)]·C_11_H_11_N_3_O_4_
*M*_r_	833.28
Crystal system, space group	Orthorhombic, *P* *b* *c* *a*
Temperature (K)	153
*a*, *b*, *c* (Å)	15.5820 (5), 20.0517 (7), 23.3082 (8)
*V* (Å^3^)	7282.5 (4)
*Z*	8
Radiation type	Mo *K*α
μ (mm^−1^)	0.68
Crystal size (mm)	0.54 × 0.44 × 0.07

Data collection	
Diffractometer	Bruker SMART APEXII
Absorption correction	Multi-scan (*SADABS*; Bruker, 2008 ▸)
*T*_min_, *T*_max_	0.712, 0.954
No. of measured, independent and observed [*I* > 2σ(*I*)] reflections	86372, 9862, 6112
*R*_int_	0.106
(sin θ/λ)_max_ (Å^−1^)	0.687

Refinement	
*R*[*F* ^2^ > 2σ(*F* ^2^)], *wR*(*F* ^2^), *S*	0.047, 0.130, 0.96
No. of reflections	9862
No. of parameters	524
No. of restraints	1
H-atom treatment	H atoms treated by a mixture of independent and constrained refinement
Δρ_max_, Δρ_min_ (e Å^−3^)	1.01, −0.68

Computer programs: *APEX2* and *SAINT* (Bruker, 2014 ▸), *SHELXS97* and *SHELXTL* (Sheldrick, 2008 ▸), *SHELXL2014* (Sheldrick, 2015 ▸) and *ORTEP-3 for Windows* (Farrugia, 2012 ▸).

## supplementary crystallographic information

### Crystal data

**Table:**

[Cu(C_24_H_24_N_8_O_6_)]·C_11_H_11_N_3_O_4_	*D*_x_ = 1.520 Mg m^−^^3^
*M**_r_* = 833.28	Mo *K*α radiation, λ = 0.71073 Å
Orthorhombic, *P**b**c**a*	Cell parameters from 8051 reflections
*a* = 15.5820 (5) Å	θ = 2.2–28.2°
*b* = 20.0517 (7) Å	µ = 0.68 mm^−^^1^
*c* = 23.3082 (8) Å	*T* = 153 K
*V* = 7282.5 (4) Å^3^	Plate, blue
*Z* = 8	0.54 × 0.44 × 0.07 mm
*F*(000) = 3448	

### Data collection

**Table:**

Bruker SMART APEXII diffractometer	6112 reflections with *I* > 2σ(*I*)
φ and ω scans	*R*_int_ = 0.106
Absorption correction: multi-scan (SADABS; Bruker, 2008)	θ_max_ = 29.3°, θ_min_ = 2.2°
*T*_min_ = 0.712, *T*_max_ = 0.954	*h* = −20→21
86372 measured reflections	*k* = −27→25
9862 independent reflections	*l* = −31→28

### Refinement

**Table:**

Refinement on *F*^2^	Primary atom site location: structure-invariant direct methods
Least-squares matrix: full	Secondary atom site location: difference Fourier map
*R*[*F*^2^ > 2σ(*F*^2^)] = 0.047	Hydrogen site location: mixed
*wR*(*F*^2^) = 0.130	H atoms treated by a mixture of independent and constrained refinement
*S* = 0.96	*w* = 1/[σ^2^(*F*_o_^2^) + (0.0736*P*)^2^ + 0.2354*P*] where *P* = (*F*_o_^2^ + 2*F*_c_^2^)/3
9862 reflections	(Δ/σ)_max_ = 0.001
524 parameters	Δρ_max_ = 1.00 e Å^−^^3^
1 restraint	Δρ_min_ = −0.67 e Å^−^^3^

### Special details

**Table:**

Geometry. All esds (except the esd in the dihedral angle between two l.s. planes) are estimated using the full covariance matrix. The cell esds are taken into account individually in the estimation of esds in distances, angles and torsion angles; correlations between esds in cell parameters are only used when they are defined by crystal symmetry. An approximate (isotropic) treatment of cell esds is used for estimating esds involving l.s. planes.

### Fractional atomic coordinates and isotropic or equivalent isotropic displacement parameters (Å^2^)

**Table:**

	*x*	*y*	*z*	*U*_iso_*/*U*_eq_	
Cu1	0.23540 (2)	0.01555 (2)	0.46535 (2)	0.01874 (9)	
O1	−0.07371 (10)	−0.09037 (9)	0.50519 (7)	0.0288 (4)	
O2	0.46419 (12)	−0.03799 (13)	0.31472 (9)	0.0542 (6)	
O3	0.43989 (13)	−0.02538 (13)	0.22515 (9)	0.0543 (6)	
O4	0.56630 (10)	0.08866 (10)	0.47203 (8)	0.0357 (4)	
O5	0.23463 (12)	0.16123 (9)	0.16939 (7)	0.0348 (4)	
O6	0.35277 (12)	0.13307 (11)	0.21053 (8)	0.0419 (5)	
N1	0.10026 (11)	−0.06399 (9)	0.41500 (7)	0.0171 (4)	
N2	0.17823 (11)	−0.04081 (9)	0.40872 (7)	0.0181 (4)	
N3	0.41581 (13)	−0.03341 (11)	0.27448 (9)	0.0293 (5)	
N4	0.36211 (11)	0.10044 (9)	0.41302 (8)	0.0197 (4)	
N5	0.27979 (11)	0.08627 (9)	0.41566 (7)	0.0180 (4)	
N6	0.27544 (13)	0.14372 (10)	0.21161 (8)	0.0244 (4)	
N7	0.18147 (11)	−0.03715 (10)	0.52509 (7)	0.0200 (4)	
N8	0.31978 (12)	0.04022 (10)	0.52296 (8)	0.0208 (4)	
C1	0.07728 (15)	−0.11055 (13)	0.57134 (10)	0.0282 (5)	
H1A	0.1254	−0.1339	0.5895	0.042*	
H1B	0.0352	−0.1432	0.5576	0.042*	
H1C	0.0500	−0.0809	0.5994	0.042*	
C2	0.10993 (14)	−0.06983 (11)	0.52124 (9)	0.0188 (4)	
C3	0.06458 (13)	−0.07253 (11)	0.46652 (9)	0.0176 (4)	
C4	−0.02728 (13)	−0.09027 (11)	0.46301 (10)	0.0203 (5)	
C5	−0.06491 (15)	−0.10480 (14)	0.40497 (10)	0.0315 (6)	
H5A	−0.1214	−0.1256	0.4096	0.047*	
H5B	−0.0269	−0.1352	0.3840	0.047*	
H5C	−0.0710	−0.0631	0.3834	0.047*	
C6	0.20627 (13)	−0.04200 (11)	0.35057 (9)	0.0175 (4)	
C7	0.29434 (13)	−0.04252 (11)	0.34078 (9)	0.0191 (4)	
H7	0.3338	−0.0463	0.3717	0.023*	
C8	0.32268 (14)	−0.03731 (12)	0.28491 (10)	0.0214 (5)	
C9	0.26778 (15)	−0.03286 (11)	0.23830 (10)	0.0224 (5)	
H9	0.2895	−0.0283	0.2004	0.027*	
C10	0.18059 (15)	−0.03522 (11)	0.24867 (10)	0.0227 (5)	
H10	0.1415	−0.0339	0.2174	0.027*	
C11	0.14957 (14)	−0.03950 (11)	0.30421 (9)	0.0204 (5)	
H11	0.0894	−0.0407	0.3108	0.025*	
C12	0.44975 (16)	0.08755 (14)	0.56354 (11)	0.0351 (6)	
H12A	0.4146	0.1030	0.5958	0.053*	
H12B	0.4891	0.1232	0.5518	0.053*	
H12C	0.4829	0.0483	0.5753	0.053*	
C13	0.39251 (14)	0.06942 (11)	0.51413 (10)	0.0213 (5)	
C14	0.41621 (13)	0.08968 (11)	0.45596 (9)	0.0204 (5)	
C15	0.50662 (15)	0.10235 (12)	0.43992 (11)	0.0257 (5)	
C16	0.52487 (15)	0.12956 (15)	0.38115 (11)	0.0377 (7)	
H16A	0.5842	0.1457	0.3795	0.057*	
H16B	0.4856	0.1665	0.3730	0.057*	
H16C	0.5168	0.0943	0.3526	0.057*	
C17	0.23544 (13)	0.10738 (10)	0.36559 (9)	0.0172 (4)	
C18	0.27775 (13)	0.11919 (11)	0.31372 (9)	0.0191 (4)	
H18	0.3385	0.1164	0.3114	0.023*	
C19	0.22975 (14)	0.13491 (11)	0.26627 (9)	0.0200 (5)	
C20	0.14114 (14)	0.14071 (11)	0.26706 (10)	0.0228 (5)	
H20	0.1098	0.1516	0.2334	0.027*	
C21	0.10001 (14)	0.12994 (11)	0.31900 (10)	0.0238 (5)	
H21	0.0393	0.1339	0.3212	0.029*	
C22	0.14640 (13)	0.11353 (11)	0.36762 (9)	0.0204 (5)	
H22	0.1172	0.1064	0.4029	0.025*	
C23	0.23529 (15)	−0.04049 (13)	0.57677 (10)	0.0249 (5)	
H23A	0.2720	−0.0808	0.5752	0.030*	
H23B	0.1984	−0.0437	0.6112	0.030*	
C24	0.29118 (15)	0.02124 (13)	0.58082 (10)	0.0260 (5)	
H24A	0.2582	0.0583	0.5982	0.031*	
H24B	0.3416	0.0120	0.6055	0.031*	
O1A	0.46165 (13)	0.22072 (15)	0.78627 (10)	0.0664 (7)	
O2A	0.28475 (13)	0.17207 (10)	0.66034 (9)	0.0457 (5)	
O3A	0.04080 (11)	0.24229 (10)	0.94118 (10)	0.0469 (5)	
O4A	0.06525 (13)	0.25224 (11)	1.03186 (10)	0.0499 (6)	
N1A	0.28478 (13)	0.20901 (10)	0.80646 (10)	0.0296 (5)	
N2A	0.33228 (13)	0.21815 (11)	0.85192 (10)	0.0332 (5)	
H2A	0.3888 (7)	0.2245 (15)	0.8469 (13)	0.051 (9)*	
N3A	0.08951 (14)	0.24565 (11)	0.98228 (12)	0.0370 (6)	
C1A	0.45036 (19)	0.21587 (17)	0.68630 (13)	0.0501 (8)	
H1A1	0.5084	0.2346	0.6880	0.075*	
H1A2	0.4142	0.2441	0.6619	0.075*	
H1A3	0.4527	0.1707	0.6703	0.075*	
C2A	0.41369 (17)	0.21322 (14)	0.74488 (13)	0.0386 (7)	
C3A	0.32060 (16)	0.20372 (12)	0.75493 (11)	0.0295 (6)	
C4A	0.25922 (17)	0.18854 (13)	0.70795 (13)	0.0341 (6)	
C5A	0.16462 (17)	0.19307 (15)	0.71948 (13)	0.0423 (7)	
H5A1	0.1413	0.2328	0.7006	0.063*	
H5A2	0.1548	0.1962	0.7609	0.063*	
H5A3	0.1360	0.1532	0.7045	0.063*	
C6A	0.29704 (16)	0.22805 (12)	0.90680 (11)	0.0277 (5)	
C7A	0.20906 (16)	0.22976 (12)	0.91655 (12)	0.0291 (6)	
H7A	0.1691	0.2227	0.8864	0.035*	
C8A	0.18222 (15)	0.24215 (12)	0.97171 (12)	0.0310 (6)	
C9A	0.23753 (18)	0.25281 (14)	1.01735 (14)	0.0367 (6)	
H9A	0.2164	0.2613	1.0549	0.044*	
C10A	0.32440 (17)	0.25058 (13)	1.00628 (13)	0.0369 (6)	
H10A	0.3642	0.2577	1.0366	0.044*	
C11A	0.35401 (16)	0.23805 (13)	0.95142 (12)	0.0330 (6)	
H11A	0.4140	0.2363	0.9443	0.040*	

### Atomic displacement parameters (Å^2^)

**Table:**

	*U*^11^	*U*^22^	*U*^33^	*U*^12^	*U*^13^	*U*^23^
Cu1	0.01805 (14)	0.02554 (15)	0.01264 (15)	−0.00435 (10)	−0.00054 (11)	0.00152 (11)
O1	0.0193 (8)	0.0433 (10)	0.0237 (10)	−0.0008 (7)	0.0055 (7)	0.0031 (8)
O2	0.0194 (10)	0.1091 (19)	0.0342 (12)	0.0037 (10)	−0.0026 (9)	0.0060 (12)
O3	0.0299 (11)	0.1047 (19)	0.0285 (12)	−0.0084 (11)	0.0138 (9)	−0.0071 (11)
O4	0.0180 (8)	0.0470 (11)	0.0420 (12)	−0.0009 (7)	−0.0038 (8)	0.0096 (9)
O5	0.0451 (11)	0.0437 (11)	0.0155 (9)	0.0029 (8)	−0.0008 (8)	0.0026 (8)
O6	0.0291 (10)	0.0713 (14)	0.0254 (11)	0.0017 (9)	0.0110 (8)	0.0074 (9)
N1	0.0152 (9)	0.0209 (9)	0.0152 (10)	−0.0009 (7)	0.0019 (7)	0.0002 (7)
N2	0.0149 (9)	0.0263 (10)	0.0132 (10)	−0.0015 (7)	0.0019 (7)	−0.0010 (7)
N3	0.0193 (10)	0.0425 (13)	0.0261 (12)	0.0009 (8)	0.0057 (9)	−0.0055 (9)
N4	0.0166 (9)	0.0229 (10)	0.0197 (10)	−0.0026 (7)	0.0023 (7)	−0.0014 (7)
N5	0.0160 (9)	0.0259 (10)	0.0122 (9)	−0.0009 (7)	0.0000 (7)	−0.0001 (7)
N6	0.0328 (12)	0.0263 (10)	0.0140 (11)	−0.0034 (8)	0.0029 (9)	−0.0022 (8)
N7	0.0187 (9)	0.0287 (10)	0.0125 (10)	−0.0021 (7)	−0.0001 (7)	0.0017 (7)
N8	0.0201 (9)	0.0283 (10)	0.0139 (10)	−0.0030 (8)	−0.0017 (7)	0.0000 (8)
C1	0.0287 (13)	0.0370 (14)	0.0189 (13)	−0.0090 (10)	0.0002 (10)	0.0064 (10)
C2	0.0189 (11)	0.0234 (11)	0.0142 (11)	0.0018 (8)	0.0021 (8)	0.0008 (8)
C3	0.0165 (10)	0.0212 (11)	0.0151 (11)	−0.0001 (8)	0.0018 (9)	0.0005 (9)
C4	0.0176 (11)	0.0249 (12)	0.0184 (12)	−0.0002 (8)	0.0006 (9)	0.0036 (9)
C5	0.0186 (12)	0.0507 (16)	0.0251 (14)	−0.0082 (11)	−0.0044 (10)	0.0038 (11)
C6	0.0196 (10)	0.0191 (10)	0.0138 (11)	−0.0022 (8)	0.0021 (9)	−0.0005 (8)
C7	0.0177 (10)	0.0251 (11)	0.0144 (11)	0.0009 (9)	−0.0019 (9)	−0.0019 (9)
C8	0.0162 (10)	0.0283 (12)	0.0196 (12)	−0.0002 (9)	0.0032 (9)	−0.0038 (9)
C9	0.0269 (12)	0.0280 (12)	0.0124 (11)	−0.0011 (9)	0.0047 (9)	−0.0022 (9)
C10	0.0253 (12)	0.0276 (12)	0.0150 (12)	0.0004 (9)	−0.0023 (9)	−0.0012 (9)
C11	0.0170 (11)	0.0245 (11)	0.0197 (12)	−0.0009 (8)	−0.0009 (9)	−0.0009 (9)
C12	0.0307 (14)	0.0470 (16)	0.0275 (15)	−0.0145 (12)	−0.0082 (11)	0.0006 (12)
C13	0.0202 (11)	0.0237 (12)	0.0200 (12)	−0.0013 (8)	−0.0007 (9)	0.0009 (9)
C14	0.0166 (10)	0.0246 (12)	0.0201 (13)	−0.0016 (8)	−0.0011 (9)	−0.0011 (9)
C15	0.0211 (12)	0.0276 (13)	0.0284 (14)	−0.0041 (9)	0.0002 (10)	−0.0010 (10)
C16	0.0212 (12)	0.0599 (19)	0.0320 (16)	−0.0085 (12)	0.0048 (11)	0.0052 (13)
C17	0.0181 (10)	0.0183 (10)	0.0152 (11)	−0.0027 (8)	−0.0004 (9)	−0.0016 (8)
C18	0.0179 (11)	0.0203 (11)	0.0191 (12)	−0.0031 (8)	0.0020 (9)	−0.0006 (9)
C19	0.0273 (12)	0.0196 (11)	0.0132 (11)	−0.0037 (9)	0.0043 (9)	0.0001 (8)
C20	0.0242 (12)	0.0240 (11)	0.0202 (13)	0.0011 (9)	−0.0042 (9)	0.0025 (9)
C21	0.0189 (11)	0.0285 (13)	0.0240 (13)	0.0024 (9)	0.0014 (9)	0.0015 (10)
C22	0.0200 (11)	0.0245 (11)	0.0168 (12)	−0.0001 (8)	0.0051 (9)	0.0020 (9)
C23	0.0241 (11)	0.0348 (13)	0.0157 (12)	−0.0046 (10)	−0.0034 (10)	0.0058 (10)
C24	0.0232 (11)	0.0399 (14)	0.0148 (12)	−0.0082 (10)	−0.0012 (10)	−0.0009 (10)
O1A	0.0253 (11)	0.125 (2)	0.0487 (15)	−0.0036 (13)	0.0007 (10)	−0.0065 (14)
O2A	0.0480 (12)	0.0499 (13)	0.0391 (13)	−0.0020 (9)	−0.0019 (10)	−0.0082 (10)
O3A	0.0217 (10)	0.0523 (13)	0.0666 (16)	0.0003 (8)	−0.0052 (10)	−0.0050 (11)
O4A	0.0394 (12)	0.0507 (13)	0.0598 (16)	−0.0008 (9)	0.0198 (11)	−0.0019 (10)
N1A	0.0268 (11)	0.0212 (10)	0.0408 (14)	0.0010 (8)	−0.0017 (10)	0.0022 (9)
N2A	0.0194 (11)	0.0390 (13)	0.0413 (15)	0.0025 (9)	−0.0025 (10)	0.0045 (10)
N3A	0.0273 (12)	0.0277 (12)	0.0561 (17)	−0.0010 (9)	0.0066 (12)	−0.0004 (11)
C1A	0.0426 (17)	0.058 (2)	0.050 (2)	−0.0103 (14)	0.0105 (15)	−0.0140 (15)
C2A	0.0309 (15)	0.0421 (16)	0.0430 (18)	0.0001 (12)	0.0060 (13)	−0.0037 (13)
C3A	0.0293 (13)	0.0230 (12)	0.0363 (16)	0.0030 (9)	0.0015 (11)	−0.0006 (10)
C4A	0.0338 (15)	0.0234 (13)	0.0451 (18)	0.0008 (10)	−0.0033 (13)	0.0007 (11)
C5A	0.0324 (15)	0.0404 (16)	0.054 (2)	−0.0016 (11)	−0.0050 (13)	−0.0117 (14)
C6A	0.0250 (12)	0.0260 (13)	0.0321 (15)	0.0032 (9)	0.0007 (11)	0.0049 (10)
C7A	0.0235 (12)	0.0246 (12)	0.0392 (16)	0.0001 (9)	−0.0047 (11)	0.0025 (10)
C8A	0.0193 (12)	0.0253 (13)	0.0483 (18)	0.0015 (9)	0.0018 (12)	0.0050 (11)
C9A	0.0367 (15)	0.0365 (15)	0.0369 (16)	0.0031 (11)	0.0011 (12)	0.0034 (12)
C10A	0.0309 (14)	0.0410 (15)	0.0388 (17)	0.0013 (11)	−0.0111 (13)	0.0055 (12)
C11A	0.0218 (12)	0.0347 (14)	0.0426 (18)	0.0023 (10)	−0.0012 (11)	0.0072 (11)

### Geometric parameters (Å, º)

**Table:**

Cu1—N7	1.9395 (18)	C15—C16	1.502 (3)
Cu1—N8	1.9434 (18)	C16—H16A	0.9800
Cu1—N2	1.9527 (18)	C16—H16B	0.9800
Cu1—N5	1.9573 (18)	C16—H16C	0.9800
O1—C4	1.221 (3)	C17—C22	1.394 (3)
O2—N3	1.207 (3)	C17—C18	1.397 (3)
O3—N3	1.220 (3)	C18—C19	1.372 (3)
O4—C15	1.225 (3)	C18—H18	0.9500
O5—N6	1.223 (2)	C19—C20	1.386 (3)
O6—N6	1.224 (3)	C20—C21	1.387 (3)
N1—N2	1.309 (2)	C20—H20	0.9500
N1—C3	1.334 (3)	C21—C22	1.384 (3)
N2—C6	1.424 (3)	C21—H21	0.9500
N3—C8	1.473 (3)	C22—H22	0.9500
N4—N5	1.315 (2)	C23—C24	1.516 (3)
N4—C14	1.326 (3)	C23—H23A	0.9900
N5—C17	1.421 (3)	C23—H23B	0.9900
N6—C19	1.470 (3)	C24—H24A	0.9900
N7—C2	1.296 (3)	C24—H24B	0.9900
N7—C23	1.469 (3)	O1A—C2A	1.230 (3)
N8—C13	1.292 (3)	O2A—C4A	1.224 (3)
N8—C24	1.471 (3)	O3A—N3A	1.224 (3)
C1—C2	1.513 (3)	O4A—N3A	1.223 (3)
C1—H1A	0.9800	N1A—N2A	1.305 (3)
C1—H1B	0.9800	N1A—C3A	1.329 (3)
C1—H1C	0.9800	N2A—C6A	1.406 (3)
C2—C3	1.459 (3)	N2A—H2A	0.898 (10)
C3—C4	1.477 (3)	N3A—C8A	1.467 (3)
C4—C5	1.503 (3)	C1A—C2A	1.481 (4)
C5—H5A	0.9800	C1A—H1A1	0.9800
C5—H5B	0.9800	C1A—H1A2	0.9800
C5—H5C	0.9800	C1A—H1A3	0.9800
C6—C7	1.391 (3)	C2A—C3A	1.482 (4)
C6—C11	1.397 (3)	C3A—C4A	1.485 (4)
C7—C8	1.379 (3)	C4A—C5A	1.501 (4)
C7—H7	0.9500	C5A—H5A1	0.9800
C8—C9	1.386 (3)	C5A—H5A2	0.9800
C9—C10	1.381 (3)	C5A—H5A3	0.9800
C9—H9	0.9500	C6A—C11A	1.382 (4)
C10—C11	1.385 (3)	C6A—C7A	1.390 (4)
C10—H10	0.9500	C7A—C8A	1.375 (4)
C11—H11	0.9500	C7A—H7A	0.9500
C12—C13	1.501 (3)	C8A—C9A	1.386 (4)
C12—H12A	0.9800	C9A—C10A	1.379 (4)
C12—H12B	0.9800	C9A—H9A	0.9500
C12—H12C	0.9800	C10A—C11A	1.382 (4)
C13—C14	1.463 (3)	C10A—H10A	0.9500
C14—C15	1.479 (3)	C11A—H11A	0.9500
			
N7—Cu1—N8	86.32 (8)	C15—C16—H16B	109.5
N7—Cu1—N2	88.42 (8)	H16A—C16—H16B	109.5
N8—Cu1—N2	157.38 (8)	C15—C16—H16C	109.5
N7—Cu1—N5	166.59 (8)	H16A—C16—H16C	109.5
N8—Cu1—N5	89.16 (7)	H16B—C16—H16C	109.5
N2—Cu1—N5	100.40 (7)	C22—C17—C18	119.0 (2)
N2—N1—C3	122.21 (18)	C22—C17—N5	118.85 (18)
N1—N2—C6	112.66 (17)	C18—C17—N5	122.12 (18)
N1—N2—Cu1	123.59 (14)	C19—C18—C17	118.63 (19)
C6—N2—Cu1	120.87 (13)	C19—C18—H18	120.7
O2—N3—O3	123.4 (2)	C17—C18—H18	120.7
O2—N3—C8	118.9 (2)	C18—C19—C20	123.5 (2)
O3—N3—C8	117.7 (2)	C18—C19—N6	117.53 (19)
N5—N4—C14	123.34 (18)	C20—C19—N6	118.9 (2)
N4—N5—C17	111.82 (17)	C19—C20—C21	117.3 (2)
N4—N5—Cu1	121.92 (14)	C19—C20—H20	121.3
C17—N5—Cu1	122.01 (13)	C21—C20—H20	121.3
O5—N6—O6	123.0 (2)	C22—C21—C20	120.7 (2)
O5—N6—C19	118.68 (19)	C22—C21—H21	119.6
O6—N6—C19	118.27 (19)	C20—C21—H21	119.6
C2—N7—C23	121.64 (18)	C21—C22—C17	120.9 (2)
C2—N7—Cu1	126.75 (15)	C21—C22—H22	119.6
C23—N7—Cu1	111.48 (13)	C17—C22—H22	119.6
C13—N8—C24	121.95 (19)	N7—C23—C24	109.98 (19)
C13—N8—Cu1	126.78 (16)	N7—C23—H23A	109.7
C24—N8—Cu1	111.27 (13)	C24—C23—H23A	109.7
C2—C1—H1A	109.5	N7—C23—H23B	109.7
C2—C1—H1B	109.5	C24—C23—H23B	109.7
H1A—C1—H1B	109.5	H23A—C23—H23B	108.2
C2—C1—H1C	109.5	N8—C24—C23	109.15 (18)
H1A—C1—H1C	109.5	N8—C24—H24A	109.8
H1B—C1—H1C	109.5	C23—C24—H24A	109.8
N7—C2—C3	119.72 (19)	N8—C24—H24B	109.8
N7—C2—C1	120.57 (19)	C23—C24—H24B	109.8
C3—C2—C1	119.46 (19)	H24A—C24—H24B	108.3
N1—C3—C2	125.46 (19)	N2A—N1A—C3A	120.5 (2)
N1—C3—C4	112.64 (18)	N1A—N2A—C6A	122.5 (2)
C2—C3—C4	121.77 (19)	N1A—N2A—H2A	118 (2)
O1—C4—C3	122.0 (2)	C6A—N2A—H2A	119 (2)
O1—C4—C5	119.6 (2)	O4A—N3A—O3A	123.6 (2)
C3—C4—C5	118.32 (19)	O4A—N3A—C8A	117.9 (2)
C4—C5—H5A	109.5	O3A—N3A—C8A	118.5 (2)
C4—C5—H5B	109.5	C2A—C1A—H1A1	109.5
H5A—C5—H5B	109.5	C2A—C1A—H1A2	109.5
C4—C5—H5C	109.5	H1A1—C1A—H1A2	109.5
H5A—C5—H5C	109.5	C2A—C1A—H1A3	109.5
H5B—C5—H5C	109.5	H1A1—C1A—H1A3	109.5
C7—C6—C11	119.83 (19)	H1A2—C1A—H1A3	109.5
C7—C6—N2	117.31 (18)	O1A—C2A—C1A	119.0 (3)
C11—C6—N2	122.79 (19)	O1A—C2A—C3A	119.1 (3)
C8—C7—C6	118.0 (2)	C1A—C2A—C3A	121.9 (3)
C8—C7—H7	121.0	N1A—C3A—C2A	122.9 (2)
C6—C7—H7	121.0	N1A—C3A—C4A	114.4 (2)
C7—C8—C9	123.2 (2)	C2A—C3A—C4A	122.7 (2)
C7—C8—N3	118.4 (2)	O2A—C4A—C3A	121.0 (2)
C9—C8—N3	118.4 (2)	O2A—C4A—C5A	119.8 (2)
C10—C9—C8	117.9 (2)	C3A—C4A—C5A	119.2 (2)
C10—C9—H9	121.0	C4A—C5A—H5A1	109.5
C8—C9—H9	121.0	C4A—C5A—H5A2	109.5
C9—C10—C11	120.6 (2)	H5A1—C5A—H5A2	109.5
C9—C10—H10	119.7	C4A—C5A—H5A3	109.5
C11—C10—H10	119.7	H5A1—C5A—H5A3	109.5
C10—C11—C6	120.3 (2)	H5A2—C5A—H5A3	109.5
C10—C11—H11	119.8	C11A—C6A—C7A	120.5 (2)
C6—C11—H11	119.8	C11A—C6A—N2A	117.0 (2)
C13—C12—H12A	109.5	C7A—C6A—N2A	122.5 (2)
C13—C12—H12B	109.5	C8A—C7A—C6A	117.2 (2)
H12A—C12—H12B	109.5	C8A—C7A—H7A	121.4
C13—C12—H12C	109.5	C6A—C7A—H7A	121.4
H12A—C12—H12C	109.5	C7A—C8A—C9A	123.8 (2)
H12B—C12—H12C	109.5	C7A—C8A—N3A	117.7 (2)
N8—C13—C14	119.7 (2)	C9A—C8A—N3A	118.4 (2)
N8—C13—C12	120.6 (2)	C10A—C9A—C8A	117.5 (3)
C14—C13—C12	119.6 (2)	C10A—C9A—H9A	121.2
N4—C14—C13	125.75 (19)	C8A—C9A—H9A	121.2
N4—C14—C15	112.74 (19)	C9A—C10A—C11A	120.4 (3)
C13—C14—C15	121.5 (2)	C9A—C10A—H10A	119.8
O4—C15—C14	122.0 (2)	C11A—C10A—H10A	119.8
O4—C15—C16	119.7 (2)	C6A—C11A—C10A	120.5 (2)
C14—C15—C16	118.2 (2)	C6A—C11A—H11A	119.7
C15—C16—H16A	109.5	C10A—C11A—H11A	119.7
			
C3—N1—N2—C6	174.16 (18)	N4—N5—C17—C22	−162.16 (19)
C3—N1—N2—Cu1	−25.0 (3)	Cu1—N5—C17—C22	40.6 (3)
C14—N4—N5—C17	175.52 (19)	N4—N5—C17—C18	20.8 (3)
C14—N4—N5—Cu1	−27.2 (3)	Cu1—N5—C17—C18	−136.36 (17)
C23—N7—C2—C3	−171.5 (2)	C22—C17—C18—C19	−1.5 (3)
Cu1—N7—C2—C3	4.0 (3)	N5—C17—C18—C19	175.48 (19)
C23—N7—C2—C1	2.7 (3)	C17—C18—C19—C20	1.0 (3)
Cu1—N7—C2—C1	178.17 (17)	C17—C18—C19—N6	−176.94 (18)
N2—N1—C3—C2	−12.0 (3)	O5—N6—C19—C18	−176.2 (2)
N2—N1—C3—C4	172.09 (19)	O6—N6—C19—C18	3.9 (3)
N7—C2—C3—N1	23.5 (3)	O5—N6—C19—C20	5.9 (3)
C1—C2—C3—N1	−150.7 (2)	O6—N6—C19—C20	−174.1 (2)
N7—C2—C3—C4	−161.0 (2)	C18—C19—C20—C21	0.1 (3)
C1—C2—C3—C4	24.8 (3)	N6—C19—C20—C21	177.9 (2)
N1—C3—C4—O1	−168.9 (2)	C19—C20—C21—C22	−0.5 (3)
C2—C3—C4—O1	15.1 (3)	C20—C21—C22—C17	0.0 (3)
N1—C3—C4—C5	7.6 (3)	C18—C17—C22—C21	1.1 (3)
C2—C3—C4—C5	−168.4 (2)	N5—C17—C22—C21	−176.0 (2)
N1—N2—C6—C7	−156.94 (19)	C2—N7—C23—C24	−156.1 (2)
Cu1—N2—C6—C7	41.6 (2)	Cu1—N7—C23—C24	27.8 (2)
N1—N2—C6—C11	26.1 (3)	C13—N8—C24—C23	−149.4 (2)
Cu1—N2—C6—C11	−135.30 (18)	Cu1—N8—C24—C23	30.5 (2)
C11—C6—C7—C8	3.0 (3)	N7—C23—C24—N8	−37.7 (3)
N2—C6—C7—C8	−173.99 (19)	C3A—N1A—N2A—C6A	176.1 (2)
C6—C7—C8—C9	−1.2 (3)	N2A—N1A—C3A—C2A	−5.2 (4)
C6—C7—C8—N3	176.08 (19)	N2A—N1A—C3A—C4A	175.8 (2)
O2—N3—C8—C7	3.4 (3)	O1A—C2A—C3A—N1A	6.4 (4)
O3—N3—C8—C7	−176.3 (2)	C1A—C2A—C3A—N1A	−171.8 (2)
O2—N3—C8—C9	−179.2 (2)	O1A—C2A—C3A—C4A	−174.6 (3)
O3—N3—C8—C9	1.1 (3)	C1A—C2A—C3A—C4A	7.2 (4)
C7—C8—C9—C10	−1.4 (3)	N1A—C3A—C4A—O2A	−168.4 (2)
N3—C8—C9—C10	−178.7 (2)	C2A—C3A—C4A—O2A	12.5 (4)
C8—C9—C10—C11	2.3 (3)	N1A—C3A—C4A—C5A	11.1 (3)
C9—C10—C11—C6	−0.5 (3)	C2A—C3A—C4A—C5A	−167.9 (2)
C7—C6—C11—C10	−2.2 (3)	N1A—N2A—C6A—C11A	−179.2 (2)
N2—C6—C11—C10	174.6 (2)	N1A—N2A—C6A—C7A	−0.9 (4)
C24—N8—C13—C14	−177.5 (2)	C11A—C6A—C7A—C8A	0.5 (3)
Cu1—N8—C13—C14	2.6 (3)	N2A—C6A—C7A—C8A	−177.7 (2)
C24—N8—C13—C12	−2.3 (3)	C6A—C7A—C8A—C9A	0.0 (4)
Cu1—N8—C13—C12	177.78 (18)	C6A—C7A—C8A—N3A	178.6 (2)
N5—N4—C14—C13	−8.6 (3)	O4A—N3A—C8A—C7A	175.2 (2)
N5—N4—C14—C15	173.19 (19)	O3A—N3A—C8A—C7A	−5.2 (3)
N8—C13—C14—N4	22.3 (4)	O4A—N3A—C8A—C9A	−6.1 (3)
C12—C13—C14—N4	−153.0 (2)	O3A—N3A—C8A—C9A	173.5 (2)
N8—C13—C14—C15	−159.6 (2)	C7A—C8A—C9A—C10A	−0.2 (4)
C12—C13—C14—C15	25.1 (3)	N3A—C8A—C9A—C10A	−178.8 (2)
N4—C14—C15—O4	−172.4 (2)	C8A—C9A—C10A—C11A	−0.1 (4)
C13—C14—C15—O4	9.2 (4)	C7A—C6A—C11A—C10A	−0.7 (4)
N4—C14—C15—C16	4.4 (3)	N2A—C6A—C11A—C10A	177.6 (2)
C13—C14—C15—C16	−174.0 (2)	C9A—C10A—C11A—C6A	0.5 (4)

### Hydrogen-bond geometry (Å, º)

**Table:**

*D*—H···*A*	*D*—H	H···*A*	*D*···*A*	*D*—H···*A*
C11*A*—H11*A*···O4*A*^i^	0.95	2.43	3.320 (3)	156
N2*A*—H2*A*···O1*A*	0.90 (1)	1.81 (2)	2.531 (3)	135 (3)
C22—H22···O1^ii^	0.95	2.27	3.207 (3)	169
C16—H16*A*···O5^iii^	0.98	2.63	3.532 (3)	154
C12—H12*B*···O3*A*^iv^	0.98	2.53	3.413 (3)	151
C11—H11···O3^v^	0.95	2.50	3.350 (3)	150
C5—H5*C*···O3^v^	0.98	2.65	3.426 (3)	137
C1—H1*B*···O3*A*^vi^	0.98	2.58	3.490 (3)	154
C1—H1*B*···O1	0.98	2.34	2.842 (3)	111

Symmetry codes: (i) *x*+1/2, −*y*+1/2, −*z*+2; (ii) −*x*, −*y*, −*z*+1; (iii) *x*+1/2, *y*, −*z*+1/2; (iv) *x*+1/2, *y*, −*z*+3/2; (v) *x*−1/2, *y*, −*z*+1/2; (vi) −*x*, *y*−1/2, −*z*+3/2.
